# Bi-Centric Independent Validation of Outcome Prediction after Radioembolization of Primary and Secondary Liver Cancer

**DOI:** 10.3390/jcm10163668

**Published:** 2021-08-19

**Authors:** Matthias Philipp Fabritius, Max Seidensticker, Johannes Rueckel, Constanze Heinze, Maciej Pech, Karolin Johanna Paprottka, Philipp Marius Paprottka, Johanna Topalis, Andreas Bender, Jens Ricke, Andreas Mittermeier, Michael Ingrisch

**Affiliations:** 1 Department of Radiology, University Hospital, LMU Munich, 81377 Munich, Germany; max.seidensticker@med.uni-muenchen.de (M.S.); johannes.rueckel@med.uni-muenchen.de (J.R.); Johanna.Topalis@physik.uni-muenchen.de (J.T.); Jens.Ricke@med.uni-muenchen.de (J.R.); Andreas.mittermeier@med.uni-muenchen.de (A.M.); 2 Department of Radiology and Nuclear Medicine, University of Magdeburg, 39120 Magdeburg, Germany; constanze.heinze@med.ovgu.de (C.H.); maciej.pech@med.ovgu.de (M.P.); 3 Department of Diagnostic and Interventional Neuroradiology, Technical University Munich, 81675 Munich, Germany; karolin.paprottka@tum.de; 4 Department of Interventional Radiology, Technical University Munich, 81675 Munich, Germany; philipp.paprottka@tum.de; 5 Department of Statistics, LMU Munich, 81377 Munich, Germany; andreas.bender@stat.uni-muenchen.de

**Keywords:** radioembolization, prediction, random survival forest, cholinesterase

## Abstract

Background: Yttrium-90 radioembolization (RE) plays an important role in the treatment of liver malignancies. Optimal patient selection is crucial for an effective and safe treatment. In this study, we aim to validate the prognostic performance of a previously established random survival forest (RSF) with an external validation cohort from a different national center. Furthermore, we compare outcome prediction models with different established metrics. Methods: A previously established RSF model, trained on a consecutive cohort of 366 patients who had received RE due to primary or secondary liver tumor at a national center (center 1), was used to predict the outcome of an independent consecutive cohort of 202 patients from a different national center (center 2) and vice versa. Prognostic performance was evaluated using the concordance index (C-index) and the integrated Brier score (IBS). The prognostic importance of designated baseline parameters was measured with the minimal depth concept, and the influence on the predicted outcome was analyzed with accumulated local effects plots. RSF values were compared to conventional cox proportional hazards models in terms of C-index and IBS. Results: The established RSF model achieved a C-index of 0.67 for center 2, comparable to the results obtained for center 1, which it was trained on (0.66). The RSF model trained on center 2 achieved a C-index of 0.68 on center 2 data and 0.66 on center 1 data. CPH models showed comparable results on both cohorts, with C-index ranging from 0.68 to 0.72. IBS validation showed more differentiated results depending on which cohort was trained on and which cohort was predicted (range: 0.08 to 0.20). Baseline cholinesterase was the most important variable for survival prediction. Conclusion: The previously developed predictive RSF model was successfully validated with an independent external cohort. C-index and IBS are suitable metrics to compare outcome prediction models, with IBS showing more differentiated results. The findings corroborate that survival after RE is critically determined by functional hepatic reserve and thus baseline liver function should play a key role in patient selection.

## 1. Introduction

Liver-directed therapies such as radioembolization (RE) play an important role as an alternative treatment option for patients with primary and secondary liver malignancies. Single-center cohorts and retrospective analyses indicate that in patients who are not eligible for surgical resection or as salvage therapy in liver-limited or liver-dominant disease, RE can prolong survival and improve quality of life [1,2,3,4,5,6,7,8,9]. Although large phase 3 randomized studies have provided disappointing results, as additional RE did not result in a significant improvement in OS, compared with standard treatment alone [10,11,12,13,14], some findings suggest that certain subgroups may benefit substantially from RE treatment [11,15].

Due to the highly variable outcomes after RE treatment and because RE is costly and potentially harmful due to severe side effects, prudent and individual patient selection plays a decisive role in therapy planning. This was recently underlined by an international multidisciplinary expert panel, which focused on individual therapy planning and made recommendations for the treatment concept [16]. A personalized dosimetry approach plays a major role, as it has been shown that on the one hand a sufficiently high tumor dose and on the other hand the damaging of healthy liver parenchyma due to too high extra tumoral doses have a strong influence on the outcome after RE [17,18,19,20]. Among other things, the panel stated that a mean absorbed dose for the nontumor liver of 40 Gy or less is considered safe [16]. However, too rigid values should be used with caution in individual therapy planning, as the functional hepatic reserve can vary greatly interindividual in patients who are often intensively pretreated. Prognostic models incorporating hepatic liver function can help to stratify patients eligible for RE and may support dosimetry planning and estimation of individual risk. In previous studies, we have developed outcome prediction models based on pretherapeutic characteristics (age, gender, primary tumor entity, hepatic tumor burden, extrahepatic disease, baseline bilirubin, and cholinesterase (CHE) levels) of a cohort of consecutive patients who had received RE due to primary or secondary liver tumor at our national center. A multivariate Cox regression identified tumor entity, hepatic tumor burden, presence of extrahepatic disease, and baseline level of CHE as independent predictors of overall survival (OS) [21]. A machine-learning approach based on random survival forests (RSF) extended and refined those results. The prognostic performance was similar to the Cox proportional hazards (CPH) model and, in addition, revealed a strong prognostic value for baseline CHE and bilirubin with a highly nonlinear influence for each parameter [22]. The aim of the present work was to validate the results of the previous studies on an independent cohort of another national center.

## 2. Materials and Methods

### 2.1. Patients

This retrospective study analyzed a previously published consecutive cohort of patients who underwent RE due to a primary or secondary liver malignancy at a single national center (center 2) in a 4-year period [23] and compared it with a previously described cohort of consecutive patients of another national center (center 1) [21]. The study was approved by the local ethics committee. Written informed consent was waived due to the retrospective nature. The selection of patients for RE was identical in both centers and based on the presence of unresectable hepatocellular carcinoma (HCC), cholangiocellular carcinoma (CCC), or hepatic metastases and lack of further chemotherapeutic options. Patients had to have a liver-predominant disease, preserved liver function, and acceptable performance status (Eastern Cooperative Oncology Group (ECOG) performance status ≤ 2).

According to the inclusion criteria of the previous studies [21,22], we included all patients with the following pretherapeutic parameters recorded one day before the first RE procedure: CHE and bilirubin levels in kU/L resp. mg/dL, sex, age, type of primary tumor, hepatic tumor burden (assessed in three categories: <25%, 25–50%, >50%) on the basis of pretherapeutic Gd-EOB-DTPA-enhanced MRI), and presence of extrahepatic disease, defined as metastatic lymph nodes or other nonlife-limiting metastases.

Pretherapeutic angiography with the application of ^99m^Tc-macroaggregated albumin was performed to evaluate the particle distribution and to determine the extent of hepatopulmonary shunting by means of SPECT examination and planar scintigraphy. Aberrant or risk vessels for potential extrahepatic microsphere deposition were coilembolized. RE was performed at a second session, as described in detail before [8,24]. Depending on tumor distribution, ^90^Y resin microspheres (SIR-Spheres^®^, Sirtex Medical, Kane Cove, Australia) were delivered selectively into the hepatic arteries in a single session (unilobar or bilobar) or in two sessions as sequential treatment of each lobe 4–8 weeks apart (sequential bilobar). The prescribed activity was calculated using the body surface area method. OS was recorded, defined as the time from the date of the first RE procedure until the death of the patient or last follow-up. Patients were censored at the time of the last follow-up if their status could not be established.

### 2.2. Statistical Analysis

#### 2.2.1. Independent Validation

An RSF [25] is an ensemble learning method that extends the random forest (RF) method [26] to deal with right-censored, time-to-event data. During training, multiple decision trees are grown with bootstrapped samples of the training data. A bootstrapped sample includes 63% of the training data on average, and the remaining part is excluded and called out-of-bag (OOB) data. At each node of a decision tree, a set of candidate variables is selected. The candidate variable that maximizes the log-rank statistic in the resulting daughter nodes is chosen for splitting. This process introduces randomness into the learning process, known to improve ensemble learning. A decision tree grows until reaching a saturation point or a predefined terminal node size. For survival data, the trained RSF returns an estimated mortality rate for each individual adjusted to the number of total events.

R, version 4.0.2 (https://www.R-project.org/, accessed on 1 July 2021) [27], was used to conduct all statistical analyses. The previously established RSF model [22] was implemented using the R package randomForestSRC [28] with the following settings: ntree = 2000, nodesize = 5, and mtry = 3. First, the RSF model was trained on the entire center 1 cohort and used to predict individual expected mortality for the independent center 2 cohort. The OOB data that were unobserved by the model during training were used to make unbiased predictions for the training data. In a second step, the RSF model was trained with the entire center 2 cohort and used to predict the outcome for the center 1 cohort and its training data. Accordingly, CPH models were implemented with the R package survival [29], trained, and used to predict outcomes.

To evaluate the prognostic performance and compare the survival models, the concordance index (C-index) [30] and the integrated Brier score (IBS) [31] were calculated. The C-index quantifies the capability of the model to correctly rank the survival times of comparable pairs based on their predictions; 1 indicates perfect sorting and 0.5 random sorting (larger values are better). The IBS calculates the integral of the mean squared distance between the predicted survival probability and the at-risk indicator evaluated at multiple time points. In contrast to the C-index, the IBS is a measure of calibration and discrimination; 0 indicates perfect prediction (smaller values are better).

#### 2.2.2. Feature Importance and Influence on Outcome

The structure of a trained RSF allows conclusions to be drawn about the importance of a variable merely by its position within the survival trees. The minimal depth (MD) [32] measures the average distance from the root to the node of the first split of the variable across all trees. The feature importance of baseline parameters age, sex, primary tumor, hepatic tumor burden, presence of extrahepatic disease, values of bilirubin, and cholinesterase was determined by measuring their MD.

Accumulated local effects (ALE) [33] and partial dependence (PD) plots [34] aim to visualize the effects of single predictor variables on the outcome. While PD plots show the marginal effect of one variable on the outcome and can be problematic for correlated features, ALE plots are an unbiased and fast alternative [35]. ALE plots were constructed for the correlated continuous variables CHE and bilirubin.

## 3. Results

### 3.1. Patients

**Figure 1:**
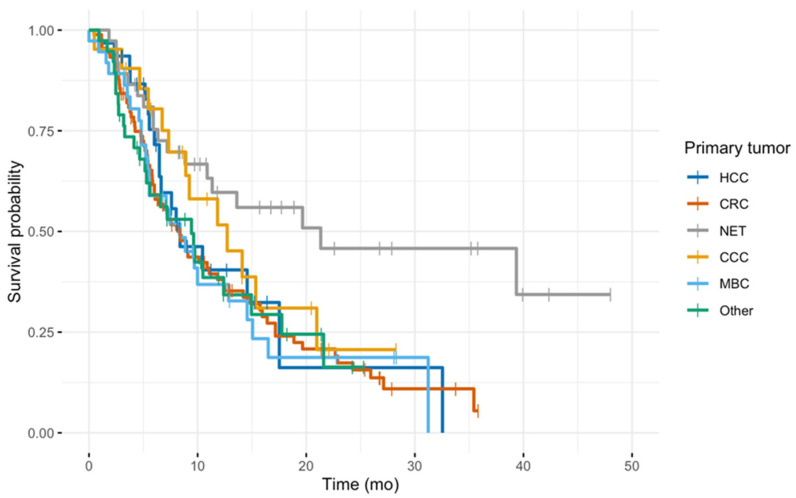
Kaplan–Meier plots for the subgroup with extrahepatic disease from center 1, color-coded by the primary tumor.

In total, 202 patients (78 female; median age 66 years) at center 2 were included in the analysis and compared with center 1 (*n* = 366). The cohorts were comparable in age, sex, as well as baseline cholinesterase values. Median bilirubin values were slightly lower in center 2 but similar in distribution. The most frequent tumor entity in both groups was metastatic colorectal cancer, followed by hepatocellular carcinoma. There were substantially fewer neuroendocrine tumors (NET) treated at center 2 (*n* = 4, 2.0%), compared to center 1 (*n* = 51, 13.9%), and less patients with presence of extrahepatic disease (*n* = 101, 50.0% vs. *n* = 253, 69.1%). Details are provided in Table 1. Observation time was 48.6 months for center 1 and 61.7 months for center 2. Median OS was higher in center 1 (11.4, 95%CI 9.7–14.2 vs. 7.9, 7.0–9.2 months) (Table 2). A subgroup analysis showed that the marked difference in OS is most likely to be explained by the high proportion of neuroendocrine tumors in center 1 (Figure 1), as these are known to have a better prognosis even in advanced stages [36].

### 3.2. Statistical Analysis

#### 3.2.1. Independent Validation

The established RSF model achieved a C-index of 0.67 for predicting center 2, comparable to center 1, in which it was trained on (0.66). The new RSF model trained on center 2 data achieved a C-index of 0.68 for center 2 and 0.66 for center 1. CPH models showed similar results, no matter which cohort was trained on and which was predicted, with C-index ranging from 0.68 to 0.72 (Table 3). IBS validation showed more differentiated results depending on which cohort was trained on, and which cohort was predicted. The established RSF model, trained on center 1, showed a better performance in predicting center 1 (0.08) than predicting center 2 (0.12). Vice versa, the same tendency was observed but more pronounced. The RSF model trained on center 2 achieved an IBS of 0.05 for predicting center 2 and 0.20 for predicting center 1. CPH models tended to show worse prediction performance, compared to RSF models when using IBS for evaluation, achieving scores from 0.08 to 0.18 (Table 3).

#### 3.2.2. Feature Importance

**Figure 2:**
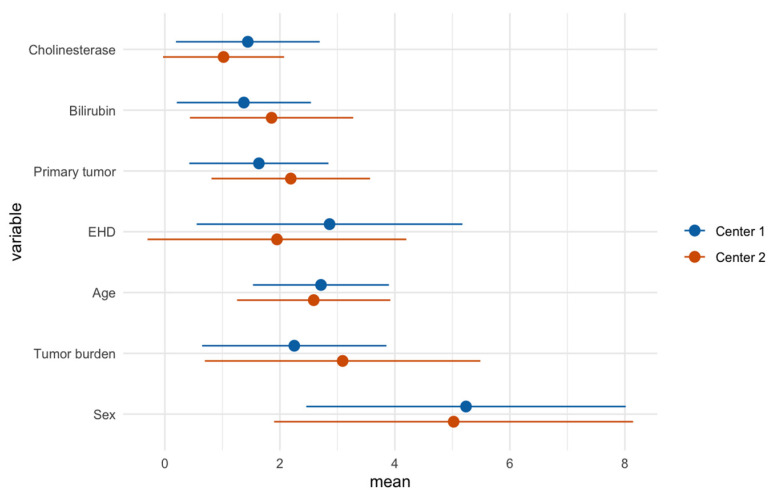
Forest-averaged minimal depth (dots) of baseline parameters with standard deviation (line). Smaller values correspond to the higher prognostic importance of variables.

In line with the results of the initial RSF (center 1), baseline CHE (forest-averaged minimal depth ± standard deviation, 1.0 ± 1.1) and bilirubin (1.9 ± 1.4) were the most important variables for survival prediction for center 2. Differences between the models were especially seen for extrahepatic disease, which had higher prognostic importance in center 2 (1.9 ± 2.3), compared to the center 1 analysis (2.9 ± 2.3). Furthermore, primary tumor and tumor burden had lower prognostic importance, compared to the center 1 analysis. Sex had the highest averaged minimal depth in both models, indicating low prognostic importance. The calculated prognostic importance levels of pretherapeutic variables for each center are illustrated and juxtaposed in Figure 2.

#### 3.2.3. Feature Importance on Predicted Outcome

**Figure 3:**
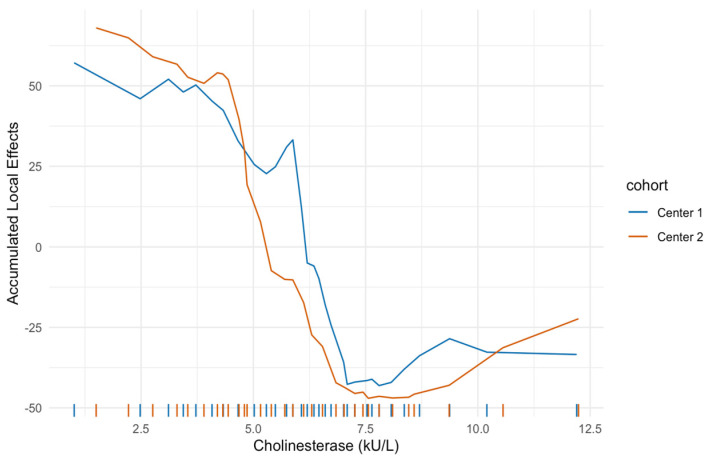
Accumulated local effect for cholinesterase. High cholinesterase levels are associated with a favorable prognosis for both cohorts. Cholinesterase levels below a threshold of 7.5 kU/L show a strong increase in expected mortality for centers 1 and 2.

**Figure 4:**
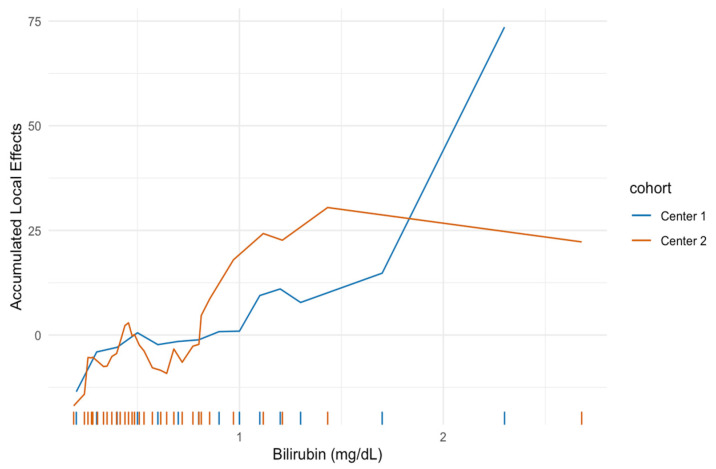
Accumulated local effect (ALE) for bilirubin. Low bilirubin levels are associated with a favorable prognosis for both cohorts. Bilirubin levels above 1 mg/dL show differences in ALE values for centers 1 and 2. However, the rug plot indicates very few observations of bilirubin for this region.

Figure 3 and Figure 4 show the ALE for pretherapeutic CHE and bilirubin levels. High CHE levels were associated with a favorable prognosis in both cohorts and showed a very similar, highly nonlinear trend. CHE levels below a threshold of 7.5 kU/L showed a strong increase in expected mortality for both centers. Low bilirubin levels were associated with a favorable prognosis in both cohorts. The bilirubin curves also exhibited an analogous course in both centers at low values, while bilirubin values above 1 mg/dL showed differences in ALE values for centers 1 and 2. However, the rug plot indicates very few observations of bilirubin for this region and therefore can only be rated to a very limited extent.

## 4. Discussion

We successfully validated the previously published RSF model to predict response to RE on an independent external cohort. The C-index showed comparable prognostic performance for all RSF models and both centers. The results were comparable to classical CPH models. IBS showed the expected differences in performance between the prediction of the training data set and an independent second data set, with the former performing better. In addition, the IBS indicated the superiority of the RSF over the CPH model when predicting the within-center cases. For the C-index, only the ranking of the predictions matters, and this depends only on the covariate effects. For the IBS, the calibration also matters, in our case, the baseline hazard, which was different in the two cohorts.

The results of the minimal depth analysis, which is a measure for the prognostic performance of a variable, were very similar in both centers. In particular, the laboratory parameters CHE and bilirubin were the most important features, ahead of tumor entity, tumor burden, or presence of extrahepatic tumor manifestation. Despite using two completely independent cohorts for estimation, the ALE plots showed a nearly identical functional shape of the covariate effect, demonstrating a nonlinear relationship between baseline CHE and outcome. CHE < 7.5 kU/L was associated with a strong increase in predicted mortality in both cohorts. The estimated bilirubin effect was also similar for both cohorts at least at lower levels. The influence of higher levels cannot be conclusively assessed because of the small number of patients with a bilirubin > 1 mg/dL in both cohorts. However, a nonlinear relationship was also evident here. A review on RE-related hepatotoxicity has shown that most RE studies use bilirubin, albumin, respectively, the ALBI score calculated from these, alkaline phosphatase, aspartate aminotransferase, alanine transaminase, and INR as pretherapeutic serum liver parameters for risk assessment and patient selection [37,38]. CHE thus far has played a rather minor role, although individual small retrospective studies suggest that it has a prognostic value in the treatment of liver tumors, including in the therapy of HCC with sorafenib or chemoembolization [39,40,41]. CHE, in combination with albumin, is one of the most important indices of the protein synthesis capacity of the liver [42]. Unlike albumin levels, which are influenced by various factors such as inflammation, chronic kidney disease, or malnutrition, CHE is less subject to fluctuations and therefore a reliable marker of liver function [43,44,45].

The fact that in two very heterogeneous cohorts with a different distribution in tumor entity and burden, as well as EHD, the liver values are more influential in the prediction of survival than any other variable, underlines how important liver function is for clinically successful RE. Possible interpretations are that the effect reflects either a late tumor stage (high tumor burden, high number of already received, potentially hepatotoxic, chemotherapies) with reduced liver function and consecutive poor prognosis and/or it is the combination of low baseline liver function and then subsequent liver parenchymal damage by RE. Regardless, when liver function is the limiting factor, RE at an advanced stage with extensive liver involvement has little chance of providing a benefit on survival but may be rather harmful. However, RE is often used as a last resort to offer patients any treatment option in liver-dominant disease after all systemic therapies have been exhausted. These patients make up large parts of study populations, which could explain the poor results of prospective RE studies. This consideration is supported by the results of the SORAMIC trial, where patients with good liver function without cirrhosis benefited most from RE [11]. Perhaps the option of complementary RE should be considered much earlier in the course of the liver-dominant disease, to achieve a better outcome of RE per se and thus on OS. Overaggressive patient selection in advanced tumor disease should be avoided and alternative systemic treatments or best supportive care considered for patients at risk. Damm et al. determined a scoring system for patient selection that comprises hepatic tumor load, CEA and CA19-9 serum levels, and Karnofsky index (TuCK-score) [46]. Late-stage patients with high tumor burden, low-performance status, and high tumor activity, as indicated by high tumor markers, will most likely not benefit from RE. Our results extend these considerations with emphasis on liver function.

Another reason for the relevance of these findings is the growing evidence that high tumor dose leads to better outcomes in RE. A post hoc analysis of the SARAH trial and a competing prospective phase 2 cohort from another group demonstrated positive correlations between survival and absorbed tumor dose in HCC [17,18]. Alsultan et al. were able to confirm these observations in patients with metastatic colorectal cancer [20]. Moreover, variations in response rates after RE suggest a medical need to optimize dose prescriptions [19]. However, high tumor doses are, in turn, associated with the risk of also damaging tumor-free liver parenchyma. Recent study results on RE, e.g., in HCC, show that liver toxicity remains a significant factor that negatively affects patient outcomes [11]. Previous reports have defined risk groups for post-RE liver toxicity even in noncirrhotic patients with metastatic disease [38]. In this context, liver function values such as CHE provide useful additional information on how far to escalate the doses and may be relevant for future treatment algorithm decision trees.

The present study is not without limitations. The validation data set of 202 patients is smaller than the initial cohort of center 1. It would certainly be interesting to test our random survival forest on larger cohorts from further institutions. Still our results, especially the results of the ALE plots, already indicate high generalizability. Furthermore, the study populations are very heterogeneous regarding the different tumor entities and thus expected survival, which doubtlessly leaves uncertainty in the reliability of the results for individual patient groups. To achieve more specific results, further subgroup analyses with even larger cohorts must be performed. Nevertheless, one of the key results of this study was to show how important general factors such as liver function are, independent of entity-specific characteristics. Another concern is that dosimetry was not considered in the analysis, although it is becoming increasingly clear how important it is for treatment response and side effects. Additionally, potentially important clinical factors such as previous treatments or renal function were not included in the model. However, the primary aim of this study was to validate the initial model with a special focus on the importance of liver function. Further refinement of these models with the inclusion of dosimetry is desirable.

## 5. Conclusions

This study successfully validated the previously published RSF model to predict survival after RE with an independent external cohort. C-index and IBS are suitable metrics to compare outcome prediction models, with IBS showing more differentiated results. Our findings indicate that survival after RE is critically determined by functional hepatic reserve, and thus, liver function plays a key role in patient selection. In particular, baseline levels of CHE may be useful to stratify patients eligible for RE and provide important guidance in the decision-making process when weighing potential treatment options.

## Figures and Tables

**Table 1:** Patient characteristics.

Characteristics	Center 1 (*n* = 366)	Center 2 (*n* = 202)
Age (y)	64 (55.7–71.0)	66 (58.0–71.0)
Sex		
Male	217 (59.3)	124 (61.4)
Female	149 (40.7)	78 (38.6)
Primary tumor		
Colorectal cancer	128 (35.0)	81 (40.1)
Hepatocellular cancer	57 (15.6)	57 (28.2)
Neuroendocrine tumor	51 (13.9)	4 (2.0)
Metastatic breast cancer	40 (10.9)	28 (13.9)
Cholangiocarcinoma	35 (9.6)	5 (2.5)
Other	55 (15.0)	27 (13.4)
Hepatic tumor burden		
25%	191 (52.2)	133 (65.8)
25–50%	140 (38.3)	53 (26.2)
50%	35 (9.6)	16 (7.9)
Extrahepatic disease		
Yes	253 (69.1)	101 (50.0)
No	113 (30.9)	101 (50.0)
Baseline liver function		
Bilirubin (mg/dL)	0.60 (0.50–0.90)	0.51 (0.35–0.78)
Cholinesterase (U/L)	6.35 (4.89–7.60)	5.88 (4.34–7.50)

Values presented are numbers (percentage) for categorical and median (interquartile range) for continuous variables.

**Table 2:** Overall survival.

Cohort	Patients	Events	Median Survival (Months)	95% CI
Center 1	366	228	11.41	9.70–14.20
Center 2	202	199	7.91	7.07–9.24

**Table 3:** Predictive performance.

Model	Training	Prediction	C-index	IBS
RSF	Center 1	Center 1	0.66	0.08
RSF	Center 1	Center 2	0.67	0.12
RSF	Center 2	Center 1	0.66	0.20
RSF	Center 2	Center 2	0.68	0.05
CPH	Center 1	Center 1	0.68	0.15
CPH	Center 1	Center 2	0.69	0.12
CPH	Center 2	Center 1	0.68	0.18
CPH	Center 2	Center 2	0.72	0.08

Concordance index (C-index) and integrated Brier score (IBS) for survival prediction with random survival forest (RSF) and Cox proportional hazards (CPH) model for center 1 and center 2.

## Data Availability

Anonymized study data are available from the corresponding author upon reasonable request.
